# Development and validation of a novel 3D-printed simulation model for open oesophageal atresia and tracheo-oesophageal fistula repair

**DOI:** 10.1007/s00383-021-05007-9

**Published:** 2021-09-02

**Authors:** Jonathan J. Neville, Carmen S. Chacon, Reza Haghighi-Osgouei, Natasha Houghton, Fernando Bello, Simon A. Clarke

**Affiliations:** 1 Department of Paediatric Surgery, Chelsea and Westminster Hospital, 369 Fulham Road, London, SW10 9NH UK; 2 Department of Surgery and Cancer, Imperial College London, London, UK; 3 Centre for Engagement and Simulation Science, Imperial College London, London, UK

**Keywords:** Simulation, 3D printing, Paediatric surgery, Oesophageal atresia, Tracheo-oesophageal fistula

## Abstract

**Background:**

The role of simulation training in paediatric surgery is expanding as more simulation devices are designed and validated. We aimed to conduct a training needs assessment of UK paediatric surgical trainees to prioritise procedures for simulation, and to validate a novel 3D-printed simulation model for oesophageal atresia and tracheo-oesophageal fistula (OA-TOF) repair.

**Methods:**

A questionnaire was sent to UK trainee paediatric surgeons surveying the availability and utility of simulation. The operation ranked as most useful to simulate was OA-TOF repair. 3D-printing techniques were used to build an OA-TOF model. Content, face and construct validity was assessed by 40 paediatric surgeons of varying experience.

**Results:**

Thirty-four paediatric surgeons completed the survey; 79% had access to surgical simulation at least monthly, and 47% had access to paediatric-specific resources. Perceived utility of simulation was 4.1/5. Validation of open OA-TOF repair was conducted by 40 surgeons. Participants rated the model as useful 4.9/5. Anatomical realism was scored 4.2/5 and surgical realism 3.9/5. The model was able to discriminate between experienced and inexperienced surgeons.

**Conclusion:**

UK paediatric surgeons voted OA-TOF repair as the most useful procedure to simulate. In response we have developed and validated an affordable 3D-printed simulation model for open OA-TOF repair.

**Supplementary Information:**

The online version contains supplementary material available at 10.1007/s00383-021-05007-9.

## Introduction

Simulation is increasingly relevant to the modern surgical trainee’s curriculum. The development of a realistic and relevant simulated operative experience enables trainees to develop technical and non-technical skills in a safe and reproducible environment [1]. This is particularly valid for paediatric surgery trainees [2]. Paediatric surgical procedures are often complex, with steep learning curves, small operating spaces, and the use of specialist equipment. The rarity of certain pathologies, and the low-volume case load make simulated procedures a vital way of gaining experience [3]. This has been compounded more recently by the coronavirus pandemic, which has resulted in fewer training opportunities for surgeons in and outside of the operating theatre [4].

Generic simulation trainers, such as laparoscopic ‘box’ trainers, allow surgeons to learn and practice core surgical skills, but are insufficient for developing the procedural and technical skills specific to paediatric surgery. This mandates the development of paediatric surgery-specific simulation resources. As of 2019, 40 paediatric surgery simulators and training courses have been described in the literature [5]. These include bench trainers, virtual reality trainers and hybrid models incorporating animal tissue. Procedures across general paediatric surgery, neonatal surgery, paediatric urology and paediatric cardiothoracics have been simulated. Despite the existence of numerous simulators, their accessibility is limited by cost, location and commercial availability [6].

The scope of paediatric surgery simulation training in the UK is currently unknown. To address this, we carried out a training needs assessment to assess the current status of simulation-based training for UK paediatric surgeons, and to identify and prioritise procedures amenable to simulation training [7]. The training needs assessment identified open oesophageal atresia and tracheo-oesophageal fistula (OA-TOF) repair as the most useful procedure to simulate. We used 3D-printing and multi-layer silicone casting techniques to develop and build an anatomically accurate OA-TOF model that can be used for both open and thoracoscopic repair. This model was then validated for open OA-TOF repair by paediatric surgeons, with the aim of creating a highly useful, anatomically realistic and cost-effective model, that could be disseminated to UK paediatric surgery trainees.

## Methods

### National simulation training needs assessment

An anonymised six-point online questionnaire was distributed to UK-based paediatric surgeons. The questionnaire was circulated through a national trainee newsletter email and by word of mouth.

Participants were asked for their current training grade and location, whether they had regular access to surgical simulation (at least once a month), and whether these resources were specific to paediatric surgery. They were asked to rate the perceived utility of simulation training to paediatric surgery on a five-point Likert scale.

Respondents were then required to rank 10 procedures in order of how useful they believed a simulation model would be to paediatric surgeons in training. Ranking each procedure first through to tenth, scored 1–10 points, respectively. The mean number of points for each procedure was compared across the participants, with the lowest mean score equating to the most popular procedure.

The ranked procedures were identified based on a review of the literature which showed few or no available simulation models [5]. The procedures ranked were: gastroschisis closure; removal of an oesophageal foreign body; open repair of OA-TOF; repair of a congenital diaphragmatic hernia; posterior sagittal anorectoplasty; pull-through for Hirschsprung’s disease; percutaneous gastrostomy (PEG) insertion; pyloromyotomy; pyeloplasty; and laparoscopic inguinal hernia repair.

### Design and construction of the simulation model

Parental informed consent was obtained to use a pre-existing computerised tomography (CT) scan of a 1 month old neonate (weighing 2.9 kg) for the purposes of developing a simulation model. The software ITK-SNAP version 3.8.0 (www.itksnap.org) was used to convert the rib cage and trachea from the neonatal CT scan to a stereolithography file for manipulation in computer-assisted design (CAD) software [8]. The CAD software Meshmixer and Fusion 360 (Autodesk Inc., CA, United States) were used to modify the rib cage and trachea, and to design the model base.

**Fig. 1:**
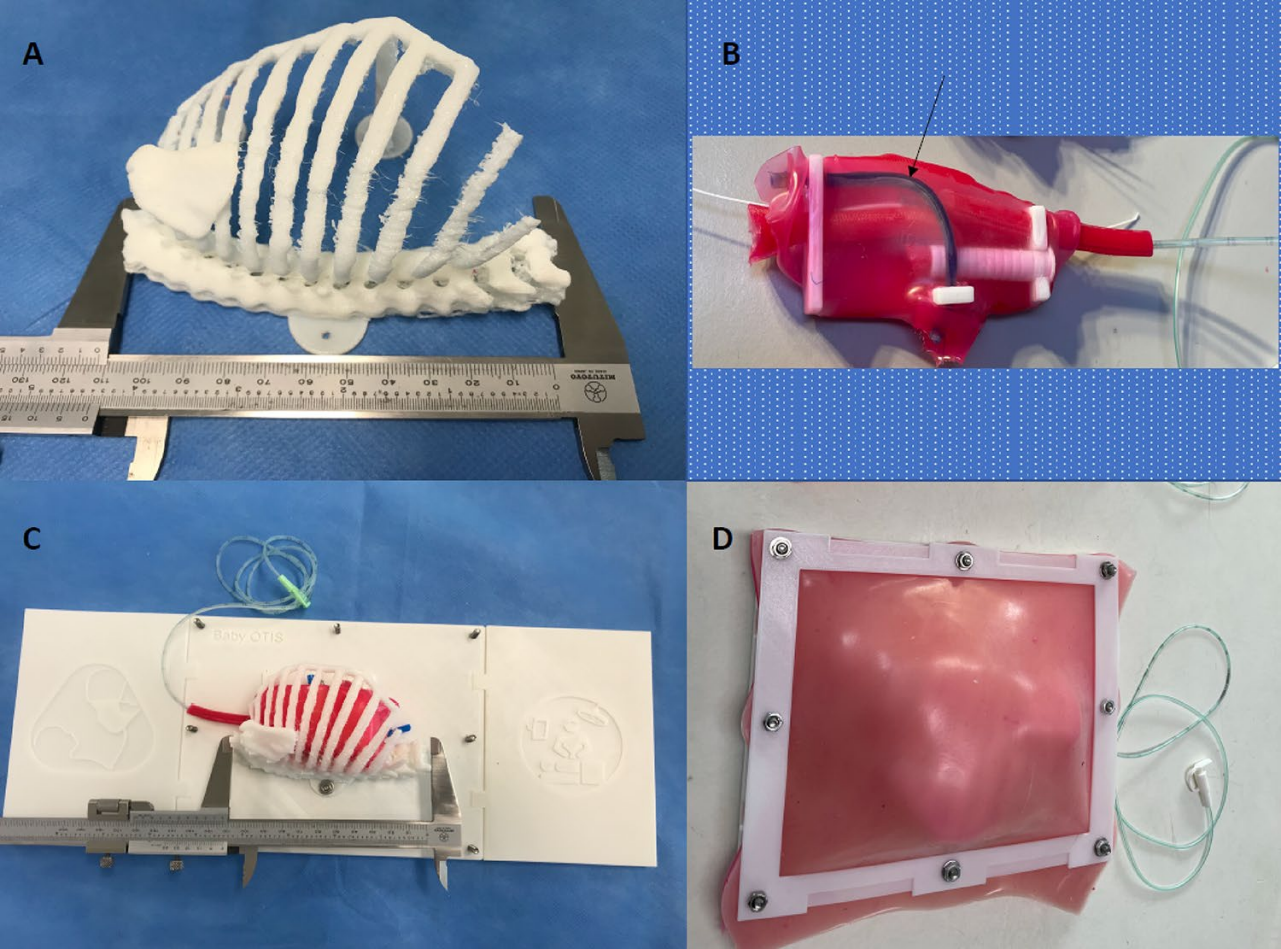
A novel 3D-printed simulation model for open oesophageal atresia and tracheo-oesophageal fistula (OA-TOF) repair. **A** 3D-printed ribcage of a 2.9 kg neonate measuring 11 cm. **B** Internal silicone structures fixed to a modular base, measuring 6 cm, to be inserted into the ribcage. Type C OA-TOF demonstrated. The azygos vein is seen in blue (arrow) and a plastic filament vagus nerve is present posteriorly. **C** Modular base inserted into the ribcage with simulated lung and a trans-anastomotic tube inserted into the upper oesophageal pouch. **D** Skin overlaid and fixed in position

The right hemithorax was isolated and orientated in the left lateral position. The ribs were reinforced at the vertebral bodies and costochondral joints with geometric shapes, and the scapula applied to the posterior ribcage. Geometric shapes were also added posteriorly and anteriorly to allow fixation of the ribcage to the model base with screws (Fig. 1A). The ribcage was 3D-printed using a Prusa i3 MK3S 3D-printer (Prusa Research, Prague, Czech Republic) and white 1.75 mm diameter flexible thermoplastic polyurethane (TPU) filament (ERYONE, Shenzhen, China). TPU filament was selected to make the ribcage flexible but strong, allowing for repeated deformation without breaking, such as during rib retraction. The trachea and model base were printed using a rigid white polylactic acid (PLA) filament (ERYONE, Shenzhen, China). The cost of these parts was approximately £100 to print.

Replaceable parts, including pleural membranes, upper and lower oesophageal pouches, the TOF and azygos vein were created using platinum-catalyzed silicone (Shore hardness 40) dyed with pigment (Smooth-On Inc., Pennsylvania, United States). The material was chosen for its likeness to tissues when handled and sutured. Silicone parts were poured and cured in 3D-printed PLA molds. A type-C TOF configuration was created (Fig. 1B). A simulated right lung was created using a household sponge. These components were applied to the 3D-printed part of the model (Fig. 1C). A double layer of softer silicone compound (Shore hardness 10), that represented skin and muscle with different colours, was fixed over the ribcage (Fig. 1D). A trans-anastomotic tube (TAT) could be passed through the upper oesophageal pouch and into the lower pouch upon completion of the anastomosis (Fig. 1B–D). The model was used to simulate > 20 open OA-TOF repairs, with only the internal silicone structures (purposefully altered during the simulated operation) requiring replacement with each use, at a cost of approximately £20 per participant. Between each participant the model could be prepared for usage again in under 20 minutes.

### Validation of the simulation model

Local ethical approval was received for the study (ICREC: 20IC5826). Data were collected using pre-designed sheets (Supplementary Material). Basic demographic information was collected prior to initiation of the simulation.

Participants were assigned to an experienced or inexperienced group based on their previous exposure to the OA-TOF procedure. Experienced participants were defined as surgeons who had performed as the primary surgeon or trained surgeons on ≥ 10 OA-TOF repairs. All participants were qualified doctors. All participants performed the procedure open.

**Fig. 2:**
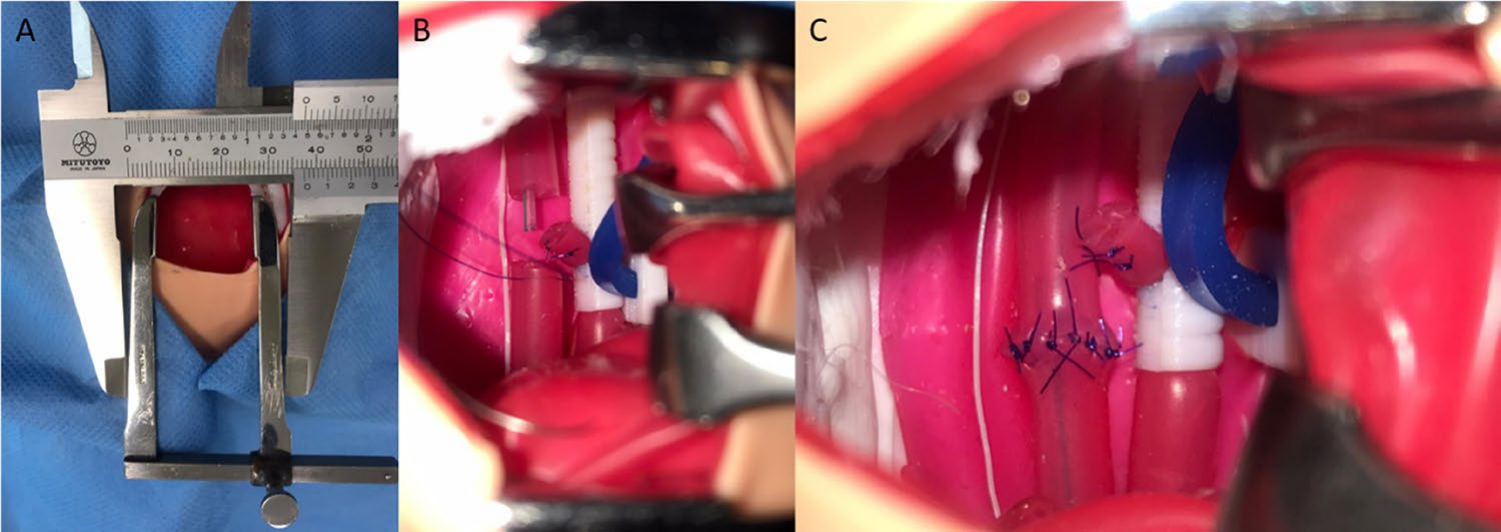
Operative views during open oesophageal atresia and tracheo-oesophageal fistula repair (OA-TOF). **A** The operative view, measuring < 3.5 cm. **B** Ligation of the TOF, and visualization of the upper and lower oesophageal pouches. The vagus is observed anteriorly (left of image), and the trachea (white) and azygos (blue) posteriorly. The trans-anastomotic tube is seen in the upper pouch. **C** Completion of the anastomosis

Construct validity was measured by evaluating the completion of the procedure by the participants. Data collection was performed by one researcher (CSC) to limit variation in scoring. Timings to complete the entire procedure, and certain key steps, were recorded. The steps timed included: completion of the right thoracotomy (Fig. 2A), dissection and visualization of the upper and lower oesophageal pouches, clipping/suturing the TOF (Fig. 2B), completion of the anastomosis (Fig. 2C), and completion of the procedure. Achievement of certain tasks were also recorded, including the passage of the TAT, and whether damage to the lung parenchyma, azygos vein, or vagus nerve occurred. The number of incorrectly or misplaced sutures was also counted, and the quality of the oesophageal anastomosis was scored on a five-point Likert scale at the end of the procedure. Other specialist equipment used during the validation included Finochietto and malleable retractors.

Content and face validity were determined using a post-simulation questionnaire. Participants were asked to rate the following characteristics of the model on a five-point Likert scale: anatomical and surgical realism, the utility of the model to paediatric surgery trainees, the utility of the model in assessing trainee skill and skill progression, the comparability of the model to a real open OA-TOF repair, and whether all trainees should have access to the model before undertaking the real procedure.

### Statistical analysis

Statistical analysis was performed in SPSS version 24 (IBM, USA). A *p* value of < 0.05 was considered significant. Data are presented as mean (± standard deviation) unless otherwise specified. Parametric variables are compared using the *t* test and non-parametric variables are compared using the Mann–Whitney *U* test. Categorical variables are compared using the Fisher’s exact test.

## Results

### National simulation training needs assessment

Thirty-four paediatric surgeons completed the online questionnaire, representing 17 UK paediatric surgery centres. Three respondents were core trainees, 19 respondents were specialist registrars ST3–ST8, four respondents were senior clinical fellows or post-CCT fellows, and eight respondents were consultants. Across all centres, 27 (79%) participants had access to surgical simulation training facilities at least monthly, but only 16 (47%) had access to paediatric surgery-specific resources. The mean perceived utility of surgical simulation for paediatric surgery trainees was high, at 4.1 (± 1.1) out of 5. Access to paediatric surgery simulation did not significantly associate with mean perceived utility (4.3 with access vs 4.1 without; *p* = 0.624). Senior paediatric surgeons (consultants, post-CCT fellows and ST8 registrars) rated the utility of simulation lower than junior surgeons, although this was not significant (3.7 vs 4.5, *p* = 0.057).

The total score for each of the 10 ranked procedures was calculated and compared (Table 1). The simulated procedure that trainees believed would be most useful to their training was OA-TOF repair (mean score 2.9 ± 2.6), followed by laparoscopic inguinal hernia repair (4.4 ± 2.6), and congenital diaphragmatic hernia repair (4.7 ± 2.4). The lowest ranked procedures were PEG insertion (6.7 ± 2.9) and oesophageal foreign body removal (6.7 ± 3.0).

**Table 1:** Simulated procedure ranking results by mean perceived benefit to UK paediatric surgery trainees

Procedure	Ranking	Mean ranking score (± SD)
Oesophageal atresia and tracheo-oesophageal fistula repair	1	2.9 (2.6)
Laparoscopic inguinal hernia repair	2	4.4 (2.6)
Congenital diaphragmatic hernia repair	3	4.7 (2.4)
Pyeloplasty	4	5.1 (2.2)
Posterior sagittal anorectoplasty	5	5.6 (3.1)
Pull-through for Hirschsprung’s disease	6	6.2 (2.7)
Gastroschisis closure	7	6.3 (2.7)
Pyloromyotomy	8	6.3 (2.1)
Percutaneous gastrostomy insertion	9	6.7 (2.9)
Oesophageal foreign body removal	10	6.7 (3.0)

*SD* standard deviation

### Validation of the simulation model

#### Participants

Forty surgeons participated in the validation of the model (Table 2). Median age was 37.5 years (interquartile range: 31.3–41.0), 21 (53%) participants were female, and 39 (98%) were right hand dominant. Twelve participants were classified as experienced, and 28 were classified as inexperienced.

**Table 2:** Model validation–comparison of the experienced and inexperienced groups

		Experienced (*n* = 12)	Inexperienced (*n* = 28)	All (*n* = 40)	*p* value
Training grade	Senior House Officer	0	3	3	< 0.001*
	Registrar	1	20	21	
	Consultant	11	5	16	
Experience {mean frequency [SD]}	Observed	53.8 (26.6)	16.1 (16.3)	27.7 (26.4)	< 0.001**
	Assisted	37.7 (14.5)	7.9 (8.7)	16.8 (17.4)	< 0.001**
	Performed	26.3 (10.2)	1.9 (2.5)	9.2 (12.7)	< 0.001**
Content validity {mean five-point Likert score [SD]}	This model is useful for paediatric surgery training	5 (0.0)	4.9 (0.4)	4.9 (0.3)	0.176**
	All trainees should have access to this model	5 (0.0)	4.7 (0.7)	4.8 (0.6)	0.279**
	The model is comparable to a real OA TOF repair	4.9 (0.3)	3.8 (1.1)	3.9 (1.0)	0.110**
	This model is useful for assessing the trainee’s skill to perform an OA TOF repair	4.3 (0.5)	4.2 (1.0)	4.3 (0.9)	0.355**
	This model is useful for assessing a trainee’s skill progress	4.5 (0.5)	4.5 (0.8)	4.5 (0.8)	0.901**
	This model should be accessible to all paediatric surgery trainees	4.5 (0.9)	4.8 (0.4)	4.8 (0.4)	0.380**
Face validity {mean five-point Likert score [SD]}	This is an anatomically realistic open OA TOF model	4.9 (0.3)	4.1 (0.7)	4.2 (0.7)	0.326**
	This is a surgically realistic open OA TOF model	4.4 (0.5)	3.9 (0.9)	3.9 (0.8)	0.586**
Construct validity (%)	Frequency successful passage of the TAT	12 (100.0)	24 (85.7)	36 (90.0)	0.297*
	Mean frequency of misplaced sutures	0.9 (1.8)	4.2 (3.7)	3.2 (3.6)	0.007**
	Mean quality of the oesophageal anastomosis	4.5 (0.5)	3.8 (0.8)	4.0 (0.8)	0.006**
	Frequency of damage to the lung	0 (0.0)	0 (0.0)	0 (0.0)	–
	Frequency of damage to the azygos vein	0 (0.0)	2 (7.1)	2 (5.0)	0.485*
	Frequency of damage to the vagus nerve	0 (0.0)	1 (3.6)	1 (2.5)	0.700*

*SD* standard deviation, *OA TOF* oesophageal atresia and tracheo-oesophageal fistula, *TAT* trans-anastomotic tube

*Fischer’s exact test

**t test

A significant difference in training grade was observed between the two groups. The experienced group contained 11 consultants and one senior registrar, whereas the inexperienced group contained 3 senior house officers, 20 registrars and 5 consultants. Operative experience was significantly higher in the experienced group compared to the inexperienced group, with a greater mean number of procedures being observed (53.8 vs 16.1, *p* < 0.001), assisted (37.7 vs 7.9, *p* < 0.001), and performed (26.3 vs 1.9, *p* < 0.001).

#### Content validity

All participants strongly believed that the model was useful for paediatric surgery training (4.9 ± 0.3), that the model should be distributed to trainees (4.8 ± 0.4), and that all trainees should have access to the model (4.8 ± 0.4). Participants less strongly believed that the model was useful for assessing a trainee’s skill to perform an OA-TOF repair (4.3 ± 0.9) and that the model was useful for assessing progress (4.5 ± 0.8). Participants scored the model lower on comparability to a real OA-TOF repair (3.9 ± 1.0). There were no significant differences in mean score for content validity between the experienced and inexperienced groups.

#### Face validity

Overall participants scored the anatomical realism of the model as 4.2 (± 0.7), with no significant difference in mean score between the experienced and inexperienced groups (4.9 vs 4.1, *p* = 0.326). Surgical realism was scored as 3.9 (± 0.8). Similarly, to anatomical realism, experienced surgeons scored the surgical realism of the model higher than inexperienced surgeons, but this was not significant (4.4 vs 3.9, *p* = 0.586).

#### Construct validity

**Fig. 3:**
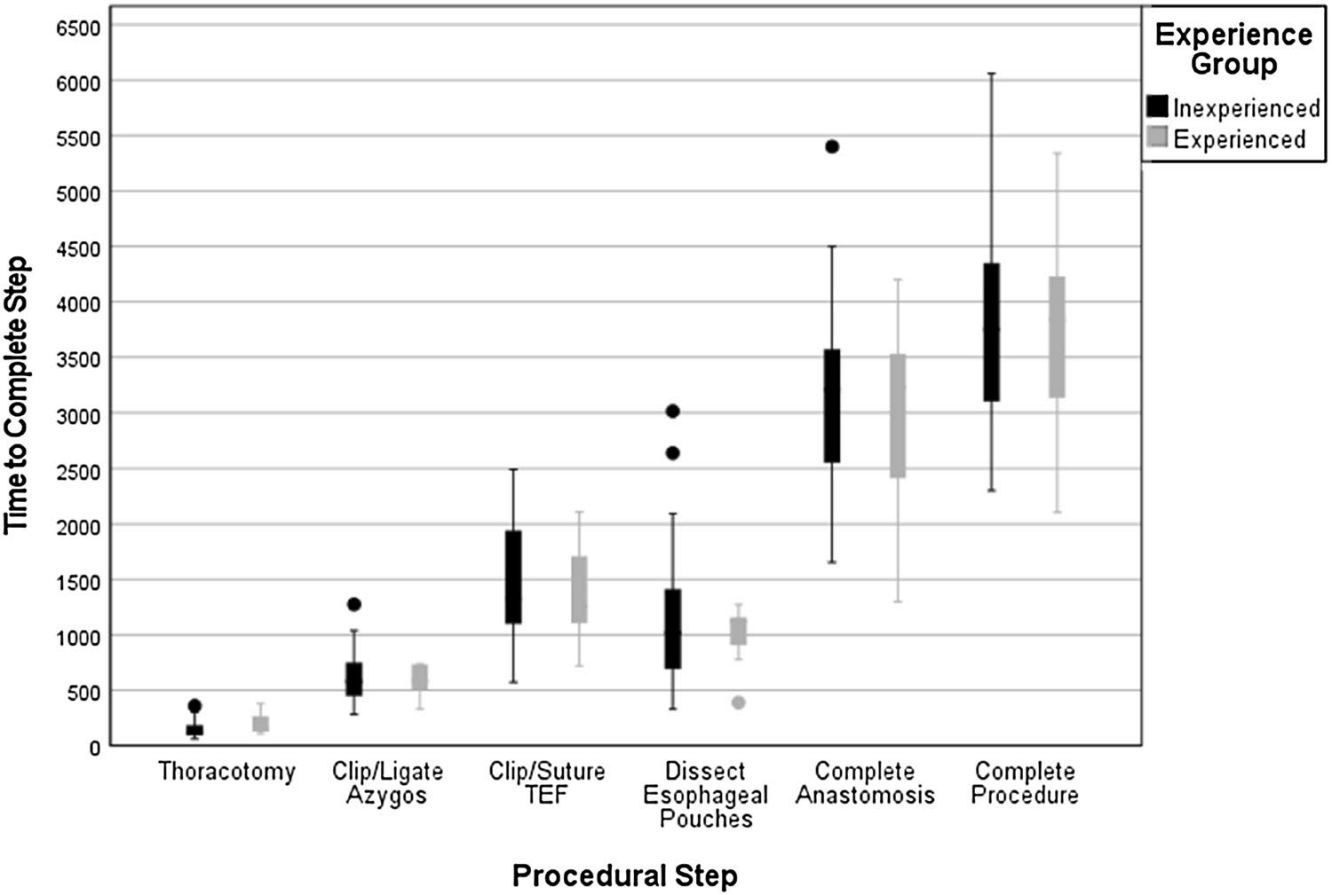
Median time in seconds to complete each key operative step in the experienced (grey) and inexperienced (black) groups. No significant difference was observed between the two groups at each stage (Mann–Whitney *U* test)

The median time to complete certain procedural steps was compared between the experienced and inexperienced groups (Fig. 3). No significant difference was observed in the median time to complete the thoracotomy (148 s vs 119 s, *p* = 0.301), to clip/ligate the azygos vein (589 s vs 580 s, *p* = 0.730), to clip/suture the TOF (1259 s vs 1327 s, *p* = 0.730), to dissect out the upper and lower oesophageal pouches (1123 s vs 1023 s, *p* = 0.730), to construct the anastomosis (3230 s vs 3208 s, *p* = 0.730), or to complete the entire procedure (3840 s vs 3753 s, *p* = 0.979).

The TAT was passed successfully more frequently in the experienced group (100 vs 86%, *p* = 0.297), and the mean quality of the oesophageal anastomosis was graded as significantly higher in the experienced group (4.5 vs 3.8, *p* = 0.007). Significantly fewer sutures were misplaced on average in the experienced group (0.9 vs 4.2, *p* = 0.006). Damage to surrounding structures occurred in the inexperienced group only, where two participants damaged the azygos vein, and one participant damaged the vagus nerve. No participants damaged lung tissue.

## Discussion

Simulation as a tool for learning is perceived as useful by paediatric surgery trainees, however, in the UK less than half have routine access to paediatric surgery-specific resources. To increase the availability of simulation to trainees, models that are affordable, reusable and clinically useful must be developed.

In this study, based on the results of a training needs assessment, we have presented a low cost, reusable 3D-printed simulation model for open OA-TOF repair. The results of the validation suggest that the model is useful for trainees, and is comparable visually and functionally to the real procedure. The model was able to successfully distinguish between experienced and inexperienced paediatric surgeons, providing objective evidence of its validity as a training tool.

### Oesophageal atresia repair simulation

To our knowledge, this is the first simulation model for open OA-TOF repair published in the literature. Other validated simulation models, of varying design and complexity, exist for thoracoscopic repair. The model we describe here can be used for both open and thoracoscopic OA-TOF repair.

Two task trainers have been developed that focus on intracorporeal knot typing in thoracoscopic OA repair. The first, by Deie and colleagues consisted of an artificial oesophagus made of polyvinyl alcohol, suspended within an artificial ribcage based on a neonatal CT scan [9]. Forty participants were asked to place a single suture across the divided oesophagus. The model was able to distinguish between experienced and inexperienced surgeons, with significantly fewer errors and manipulations, and a shorter time to complete the task, being observed in the experienced group. Similarly, Bökkerink and colleagues described a low-cost procedural model for OA repair without TOF, consisting of two rubber balloons separated on a suture pad inside a laparoscopic box trainer [10]. It was perceived as useful as a training tool, and scored 3.8/5 (± 0.8) for visual realism and 3.6/5 (± 0.8) for tissue haptics. Both models represent useful and low-cost task trainers for OA repair anastomosis, but lack other steps in the procedure. Task trainers such as these are beneficial, because parts can be cheaply replaced, and the model can used multiple times. Our model was constructed with reusability in mind. It contains a modular insert that can be removed, allowing for the internal silicone structures used in the procedure to be easily replaced. After a participant completes a full OA-TOF repair, the model can be prepared for a subsequent operation within minutes. Reusable, and therefore, cost effective, models are necessary if simulation is to become more accessible to trainees.

Barsness and colleagues created a hybrid model for thoracoscopic OA-TOF repair using a 3D-printed term neonate ribcage combined with fetal bovine tissue [11, 12]. Twenty surgeons undertook validation, and the model scored highly in all validity domains tested. Similarly to our study, no significant difference in the time to complete the operation was observed between the experienced and inexperienced groups. Although, fewer inexperienced surgeons had completed an anastomosis at the end of the procedure. Due to cost, availability, hygiene and ethical considerations, Barsness and colleagues later updated the model by replacing the animal tissues with a silicone insert [13]. Forty-seven surgeons used the model. Validity scores remained high, but were notably less than for the hybrid model.

Maricic and colleagues constructed a model designed to simulate a 3 kg infant using polyvinyl chloride plastic tubing for the ribcage and latex tubular balloons for the oesophageal pouches [14]. Thin plastic film was used to simulate pleural membranes and further rubber structures were used to simulate the azygos vein, vagus nerve and lung. Of the 39 participants who validated the model for thoracoscopic repair, 94% considered the model to have a high degree of anatomical realism, 88% agreed the model had a high degree of surgical realism, and 87% believed that the model could be used to generate the skills required to perform an OA-TOF repair. The model could also differentiate between experienced and inexperienced participants for time to complete the procedure and quality of the anastomosis.

Wells and colleagues constructed a thoracoscopic OA-TOF repair simulator using a 3D-printed ribcage, and silicone skin and OA-TOF insert [6]. Similarly, to our own model, they accurately constructed the ribcage based on a real neonatal CT scan, to ensure appropriate dimensions and a small operative space. The authors updated the model through two validations and observed improving outcomes with each development iteration. Validating the third version with 10 participants, the model scored highly in relevance to practice and value to training, but less in realism.

Similar scores for anatomical and surgical realism were obtained in our study (4.2/5 and 3.9/5), as in Wells et al*.* (3.6/5 and 3.6/5), and Barsness et al*.* (4.1/5 and 4.2/5) [6, 13]. Higher scores for realism were achieved with the hybrid model, using animal tissue, highlighting the trade-off between using animal tissue for realism, and silicone equivalents for ethical, cost and hygiene reasons. However, the lack of anatomical realism in these studies did not detract from the perceived utility of the model. Procedurally accurate ‘medium-fidelity’ models incorporating silicone parts, such as the model we have presented here, have major cost and distribution benefits over more expensive high-fidelity models.

### 3D-printing in surgical simulation

The use of 3D-printing technology to build simulation models has numerous benefits. Generally, 3D printing is cost-effective and easily scalable. After the initial investment in a 3D printer, which can be purchased for £200–1000, our entire model could be manufactured for under £100. Because of the reusability of many of the model’s parts, the cost to perform a simulated procedure per participant is £20.

The combined use of CAD and 3D-printing facilitates the production of simulation models recreated from real patient imaging data. Structures can be designed to exactly replicate patient anatomy and models can be built to replicate variant patient anatomy. Skill development can, therefore, occur in an appropriately scaled environment, which is necessary in paediatric surgery where the operative field is often small. 3D printing also affords greater variability in materials used throughout the model, allowing for materials to be selected based on similarity to real biological structures. 3D-printed models also negate the cost and ethical implications of using animal tissues.

### Study limitations

The external validity of the training needs assessment is limited somewhat for locations outside of the UK. The availability of simulation training resources is likely to differ considerably between countries. However, the validity of trainee perceptions of simulation training, and the ranking of the useful procedures is still expected to be relevant, since rare conditions and steep learning curves in paediatric surgery are global issues.

Forty participants validated the model, a sample size comparable to other validation studies of similar models [5, 6, 11]. The use of participant experience to validate simulation models has been used previously in the literature, but significant variation exists in the definition of experienced and inexperienced candidates making comparisons between studies difficult [7]. Furthermore, inaccuracies in self-reported experience levels are likely to exist [15]. However, in this study, we observed a significant difference in operative experience between the two groups and therefore, we can infer that the better performance of experienced participants observed is due to a greater level of skill.

Although the model presented here scored high in content and face validity, the transferability of the skills practiced on the model to the operating theatre, and the impact of this training on patient outcomes is unknown. This represents a worthwhile area of further study.

### Implications for training and future directions

The results of the training needs assessment presented here will enable the development of highly useful simulation models, minimize duplications of effort, and focus resources. Demand also exists for models simulating laparoscopic inguinal hernia repair, congenital diaphragmatic hernia repair, and pyeloplasty. Further model development should focus on these procedures.

**Fig. 4:**
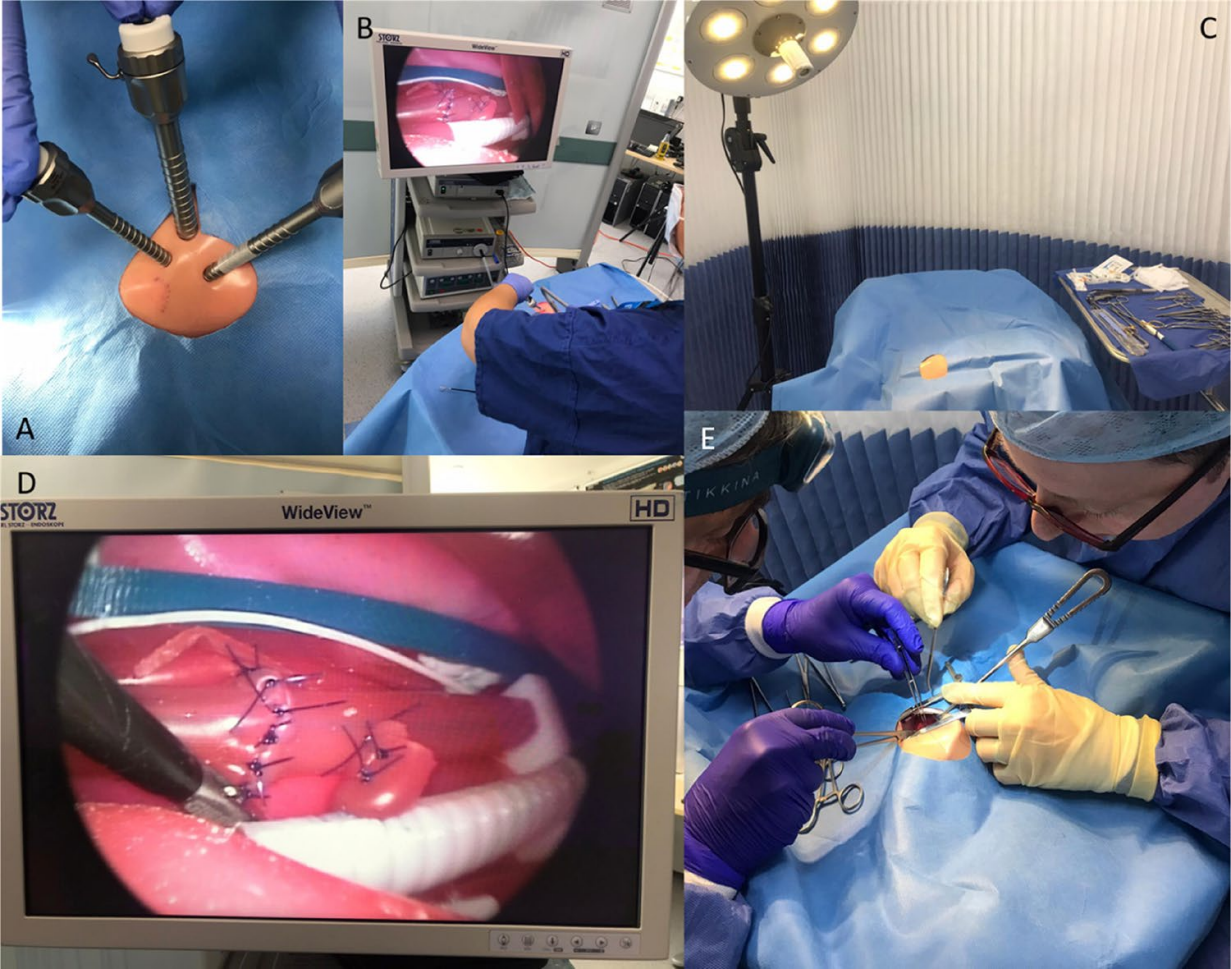
**A**, **B**, **D** Set up and use of the model for thoracoscopic repair of oesophageal atresia and tracheo-oesophageal fistula. **C**, **E** Realistic operating theatre scenarios allow the model to be used for non-technical and human factors simulation training

Paediatric surgery-specific simulation models should be made widely available to trainees on and outside of formal courses, to allow for frequent practice. Simulation training can assist in overcoming the current shortfall in trainee caseloads caused by the coronavirus pandemic [4]. To facilitate this, models should be developed that are cost effective and scalable; 3D printing is likely to be a useful tool to enable this to occur. We hope to disseminate this model to all UK paediatric surgery centres through educational grants, and to validate the model for thoracoscopic OA-TOF repair (Fig. 4A, B, D). We also plan to use the model for non-technical skills and human factors training in realistic operating theatre scenarios (Fig. 4C, E). Further studies are required to measure skill transfer from simulated environments into the operating theatre to determine whether simulation training improves clinical outcomes. Once proven to have high content, face and construct validity, a more detailed clinical outcome study can be undertaken using this model to prove objective utility.

## Conclusion

Paediatric surgeons in the UK perceive simulation as useful to their training, but access to paediatric surgery-specific resources is limited. We have identified the surgical procedures that paediatric surgeons believe would be most useful simulated, and further work should focus on building and validating models that meet this need. We created and validated a novel, cost-effective simulation model for open OA-TOF repair, which both experienced and inexperienced paediatric surgeons found to be highly realistic and useful for skill acquisition.

## Supplementary Information

Below is the link to the electronic supplementary material.

Supplementary file1 (DOCX 15 KB)

## Abbreviations

OA-TOF: Oesophageal atresia and tracheo-oesophageal fistula
CAD: Computer-assisted design
TPU: Thermoplastic polyurethane
PLA: Polylactic acid
TAT: Trans-anastomotic tube
