# Supplementary material for: Development and validation of a novel 3D-printed simulation model for open oesophageal atresia and tracheo-oesophageal fistula repair
Source: Pediatr Surg Int. 2021 Sep 2;38(1):133–41. doi: 10.1007/s00383-021-05007-9 (PMC8412403; doi:10.1007/s00383-021-05007-9)
Supplement: Supplementary file 1 — Supplementary file1 (DOCX 15 KB) [file 383_2021_5007_MOESM1_ESM.docx]

**Supplemental Information**

| **Open Technique** | |
| --- | --- |
| **Participant Information**  *Please complete or delete as appropriate* | |
| ID Number |  |
| Age |  |
| Gender | Female / Male |
| Dominant Hand | Left / Right |
| Training Stage |  |
| Number of OA-TOF repairs **observed**  *(Best estimation please, state if unknown or >50)* |  |
| Number of OA-TOF repairs **assisted** |  |
| Number of OA-TOF repairs **performed** |  |
| **Content Validity**  *Score your agreement out of five – five is strongly agree and one is strongly disagree* | |
| This model is useful for paediatric surgery training |  |
| All trainees should have access to this model before undertaking a real OA-TOF repair |  |
| This model is comparable to a real OA-TOF repair |  |
| This model is useful for assessing the trainee’s skill to perform an OA-TOF repair |  |
| This model is useful for assessing a trainee’s skill progress |  |
| This model should be accessible to all paediatric surgery trainees |  |
| **Face Validity**  *Score your agreement out of five – five is strongly agree and one is strongly disagree* | |
| This is an anatomically realistic open OA-TOF model |  |
| This is a surgically realistic open OA-TOF model |  |
| **Construct Validity** | |
| Time to complete thoracotomy | Seconds |
| Time to clip/ligate azygos vein | Seconds |
| Time to clip/suture tracheo-oesophageal fistula | Seconds |
| Time to dissect upper and lower oesophageal pouches | Seconds |
| Time to complete anastomosis | Seconds |
| Time to complete entire procedure | Seconds |
| Successful passage of trans-anastomotic tube | Yes / No |
| Quality of oesophageal anastomosis  *(ranked out of five – five is very good and one is very poor)* |  |
| Frequency of misplaced sutures |  |
| Damage to the lung | Yes / No |
| Damage to the azygos vein | Yes / No |
| Damage to the vagus nerve | Yes / No |
| **Further Comments/Feedback:** | |
|  | |

Supplementary Material: Participant data collection sheet used during validation.
